# Accuracy of breeding values for production traits in turkeys (*Meleagris gallopavo*) using recursive models with or without genomics

**DOI:** 10.1186/s12711-021-00611-8

**Published:** 2021-02-16

**Authors:** Emhimad A. Abdalla, Benjamin J. Wood, Christine F. Baes

**Affiliations:** 1 Centre for Genetic Improvement of Livestock, University of Guelph, Guelph, ON Canada; 2 School of Veterinary Science, University of Queensland, Gatton Campus, Gatton, QLD Australia; 3 Hybrid Turkeys, C-650 Riverbend Drive, Suite C, Kitchener, Canada; 4 Institute of Genetics, Vetsuisse Faculty, University of Bern, Bern, Switzerland

## Abstract

**Background:**

Knowledge about potential functional relationships among traits of interest offers a unique opportunity to understand causal mechanisms and to optimize breeding goals, management practices, and prediction accuracy. In this study, we inferred the phenotypic causal networks among five traits in a turkey population and assessed the effect of the use of such causal structures on the accuracy of predictions of breeding values.

**Methods:**

Phenotypic data on feed conversion ratio, residual feed intake, body weight, breast meat yield, and walking score in addition to genotype data from a commercial breeding population were used. Causal links between the traits were detected using the inductive causation algorithm based on the joint distribution of genetic effects obtained from a standard Bayesian multiple trait model. Then, a structural equation model was implemented to infer the magnitude of causal structure coefficients among the phenotypes. Accuracies of predictions of breeding values derived using pedigree- and blending-based multiple trait models were compared to those obtained with the pedigree- and blending-based structural equation models.

**Results:**

In contrast to the two unconditioned traits (i.e., feed conversion ratio and breast meat yield) in the causal structures, the three conditioned traits (i.e., residual feed intake, body weight, and walking score) showed noticeable changes in estimates of genetic and residual variances between the structural equation model and the multiple trait model. The analysis revealed interesting functional associations and indirect genetic effects. For example, the structural coefficient for the path from body weight to walking score indicated that a 1-unit genetic improvement in body weight is expected to result in a 0.27-unit decline in walking score. Both structural equation models outperformed their counterpart multiple trait models for the conditioned traits. Applying the causal structures led to an increase in accuracy of estimated breeding values of approximately 7, 6, and 20% for residual feed intake, body weight, and walking score, respectively, and different rankings of selection candidates for the conditioned traits.

**Conclusions:**

Our results suggest that structural equation models can improve genetic selection decisions and increase the prediction accuracy of breeding values of selection candidates. The identified causal relationships between the studied traits should be carefully considered in future turkey breeding programs.

## Background

The number of traits included in genetic evaluation programs has increased steadily over time. Hence, multivariate linear mixed models (MTM), e.g. [1, 2], are increasingly important in animal breeding. Although MTM have been successfully used to study the genetic and environmental relationships between phenotypes, they do not infer the phenotype networks that describe the interrelationships that are generally present in biological systems. Models that account for recursiveness and feedback between traits, such as structural equation models (SEM) [3, 4],  can be used to evaluate simultaneous relationships that exist between phenotypes. Knowledge about cause-and-effect mechanisms that underlie interrelationships between environmental factors, management practices, animal physiology, and performance outcomes is crucial to explore the behavior of complex systems and can contribute to improving management practices and multi-trait selection strategies in livestock.

A phenotypic correlation between two traits, y_1_ and y_2_, can be due to a direct effect of one on the other or to extraneous (confounding) variables that jointly affect both traits, among other possibilities. Defining the causal structure allows prediction of the effect of interventions (e.g., management practices) applied to each trait. When, for instance, y_1_ affects y_2_ while y_2_ does not affect y_1_, an intervention on y_1_ will cause changes on y_2_, but not conversely. Structural equation models have been used in many fields including genetics, economics, psychometrics, social statistics, and biological sciences [5]. From their studies on bovine milk fatty acid traits and meat quality traits of Wagyu beef, Bouwman et al. [6] and Inoue et al. [7], respectively, compared MTM and SEM and observed differences in estimates of parameters between them.

Similar to other species, MTM has been widely applied to assess the associations between production, reproduction and welfare traits in turkeys. Quinton et al. [8], examined the connections between survival and fitness in turkeys using MTM and concluded that selection for growth may decrease survival and traits related to walking ability. Their suggestion was to perform a multiple trait selection program for growth, survival, walking ability, hip and leg structures. Analyzing walking ability, breast meat yield, and feed efficiency traits, Abdalla et al. [9] reported unfavorable genetic correlations of walking ability with body weight and breast meat yield. Other applications of MTM for turkeys were presented by Emamgholi Begli et al. [10] to estimate phenotypic and genetic correlations between clutch and broodiness traits, along with body weight and other conventional egg production traits. Similar to previous literature, the authors reported an unfavorable genetic correlation between egg production and body weight and suggested that including clutch and pause length traits in the selection index may reduce broodiness while increasing egg number.

The objectives of this study were: (1) to search for causal structures among body weight, walking ability, breast meat yield, and two feed efficiency traits in a turkey breeding line; (2) to fit a SEM based on the uncovered causal structures to quantify the relationships between the traits; and (3) to compare MTM and SEM in terms of prediction accuracy of breeding values based on pedigree-based (BLUP) and single-step blending (ssGBLUP) animal models.

## Methods

### Phenotypic data

Data used in this study were previously described [9] and further details are in Table 1. Briefly, the data were provided by Hybrid Turkeys, Kitchener, Canada, from 10 generations of a turkey breeding line from birds hatched between 2009 and 2017. Feed conversion ratio (FCR; N = 5619), residual feed intake (RFI; N = 5619), and breast meat yield (BMY; N = 9634) were recorded on male birds, whereas body weight (BW; N = 170,844) and walking score (WS; N = 170,844) were recorded on both males (N = 99,832) and females (N = 71,012). A standard feeding system with group housing and shared feeders and drinkers was used until 15 weeks of age. From this point on and until 19 to 20 weeks of age, a real-time automated system was used to monitor individual feed intake in males. With this system, each bird was identified during each visit to the feed station and the weight of the feeder in addition to the body weight of the bird were taken using a scale [11]. FCR was calculated as total feed intake divided by weight gain, while RFI was obtained as the residual of a multiple regression of observed feed intake on metabolic mid-weight and body weight gain [12]. Based on walking ability, a subjective WS ranging from 1 to 6 was given to each male and female at 18 and 20 weeks of age, respectively. Birds with better walking ability received a higher WS. Males were slaughtered at 21 or 22 weeks to obtain BMY, which was calculated as:

$${\text{BMY}} \, \text{=} \, \frac{\text{Breast meat weight}}{\text{Live body weight at slaughter}}\times \, {100}.$$

**Table 1:** Descriptive statistics of the analyzed dataset including number of records, mean, standard deviation, training and validation subsets for different production and fitness traits in a turkey line

Trait	Number	Mean	Std	Training		Validation	
				Genotyped	Not genotyped	Genotyped	Not genotyped
Feed conversion ratio (kg/kg)	5592	2.58	0.39	2307	2711	110	464
Residual feed intake (kg)	5592	0.00	2.51	2307	2711	110	464
Body weight (kg)	170,844	17.50	5.32	13,862	139,061	1138	16,783
Breast meat yield (%)	9634	24.37	2.33	843	7877	136	778
Walking score (1 to 6)	170,844	2.10	0.86	13,862	139,061	1138	778

### Genomic data

Genotypes from a proprietary 65 K single nucleotide polymorphism (SNP) panel (65,000 SNPs; Illumina, Inc.) were available for a subset of the animals as reported in Table 1. SNPs that were located on the sex chromosome, those that deviated significantly from Hardy–Weinberg proportions $(P<1 \times {10}^{-8}),$ and SNPs with a minor allele frequency lower than 5% or with a call rate lower than 90% were removed. The number of SNPs that remained for analysis was 53,455. All genotyped animals had a call rate higher than 90% and all were included in the study.

### Structural equation modeling (SEM)

Three main steps are required to perform SEM [5, 13]:

(1) Draw samples from the posterior distribution of the covariance matrices of residuals from a multiple trait model.

(2) Apply the inductive causation (IC) algorithm to query about the statistical independence between two traits. In each query, the IC performs the following:

- Compute the posterior distribution of residual partial correlation between two traits for each sample that is drawn from the posterior distribution of covariance matrices of residuals.
- Obtain the highest posterior density (HPD) for the posterior distribution of the residual partial correlation.
- Two traits are declared conditionally dependent if the HPD interval contains 0.

(3) Fit the SEM based on the selected causal structure.

## Multiple trait model (MTM)

### Pedigree-based best linear unbiased prediction (PBLUP)

To estimate genetic and residual (co)variance components using pedigree relationships, the following multiple trait linear mixed model was fitted:

$$\mathbf{y}\ \text{=}\ \mathbf{ Xb }\ \text{+}\ \mathbf{ Zu }\ \text{+}\ \mathbf{ e},$$

where, $\mathbf {y}$ is a vector of observations of FCR, RFI, BMY, BW and WS, sorted by animals; $\mathbf {b}$ is a vector with the systematic effects of hatch week-year for all traits and sex for BW and WS; $\mathbf {u}$ is a vector of additive genetic effects, distributed as $\mathbf{u}\sim N(\mathbf0, \mathbf{A}\otimes\mathbf{G}),$ where $\mathbf{A}$ is the numerator relationship matrix, derived by including inbreeding coefficients, and $\mathbf{G}$ is the additive genetic variance–covariance matrix among traits; $\mathbf {e}$ is a vector of residual effects, distributed as $\mathbf{e}\sim N(\mathbf0, {\sum }_{\mathrm{i}}^{+}{\mathbf{E}}_{iy})$ where ${\mathbf{E}}_{iy}$ indicates a ${m}_{i}\times {m}_{i}$ matrix corresponding to the trait phenotypes that were available for animal $i$, and ${m}_{i}$ is the number of trait phenotypes available for animal $i$; $\mathbf {X}$ and $\mathbf {Z}$ are incidence matrices for the respective fixed and random effects.

### Single-step genomic best linear unbiased prediction (ssGBLUP)

The same model outlined above for PBLUP was used for ssGBLUP, except that the $\mathbf{A}$ matrix was replaced by the $\mathbf{H}$ matrix, which is a combined pedigree and genomic relationship matrix [14]. For both models (PBLUP and ssGBLUP), a Markov chain Monte Carlo (MCMC) method based on Gibbs sampling was used to estimate the joint posterior distribution to derive posterior means and standard deviations of parameters of interest. Using the software THRGIBBS1F90 [15], a single chain of the Gibbs sampler was run, with the first 100,000 samples discarded as burn-in. Thereafter, samples were saved every 50 iterations, resulting in 20,000 samples. Convergence was assessed by visual inspection of trace plots of posterior distributions and Geweke’s diagnostic [16]. The latter ranged from − 0.20 to 0.24 for all variance–covariance parameters.

### Inductive causation (IC) algorithm

The IC algorithm can be used to explore the full space of possible networks among traits [17]. When two traits are genetically correlated, residual (co)variances are generated to correct for the confounding among them. The IC algorithm performs a series of statistical decisions based on partial correlations between traits. First, IC computes the posterior distribution of the residual partial correlation between two traits at each sample drawn from the posterior distribution of covariance matrices of residuals derivative from MTM (PBLUP or ssGBLUP). Then, the HPD for the posterior distribution of residual partial correlation is obtained. The process to declare that two traits are conditionally dependent or not can be divided into three main steps: (1) two traits are considered connected with an undirected link (e.g., y_1_–y_2_) if the partial correlations among them conditional on every possible set of the remaining traits are declared different from 0; (2) if the two traits are nonadjacent but share a common adjacent trait (e.g., y_1_ and y_3_ in y_1_–y_2_–y_3_) and their partial correlations are conditionally non-null on all possible subsets of the remaining traits that contain the adjacent trait (y_2_ in this case), arrowheads pointing to the common adjacent trait can be added (i.e., y_1_ → y_2_ ← y_3_), which is known as an unshielded collider; and (3) without creating a new unshielded collider or cycle, as many undirected links as possible are oriented based on the partially oriented graph obtained in step 2. Declaration of partial correlations to be null or not was based on the HPD intervals, i.e. a correlation was declared to be null if the HPD contained the value 0. To evaluate the structure sensitivity [5, 13], HPD intervals of 85, 90, and 95% were applied. The IC analysis was carried out by using a previously described software program [17] written in R [18].

### Structural equation models

Based on the causal network inferred by the IC algorithm, two SEM models were fitted: the structural equation PBLUP (SEPBLUP) and the structural equation ssGBLUP (SEssGBLUP). For SEPBLUP, the model was:

$${\mathbf{y}} = (\sum_{\text{i}}^{+} \varvec{\Lambda}_{iy}\mathbf{)y + X}{\mathbf{b}}^{*}\mathbf{ + Z} {\mathbf{u}}^{*}\text{ + }{\mathbf{e}}^{*},$$

where $\mathbf {y}$ is a vector of observations of FCR, RFI, BMY, BW, and WS sorted by animal; $\mathbf {X}$, ${\mathbf{b}}^{\boldsymbol{*}}$, $\mathbf{Z}$, ${\mathbf{u}}^{\boldsymbol{*}}$, ${\mathbf{e}}^{\boldsymbol{*}}$, hold similar meanings to those for the MTM described above, except that the vectors here serve as systematic and random effects that directly affect each trait and that are not mediated by other traits [4, 5, 17]. $\varvec{\Lambda}_{iy}$ is a ${m}_{i}\times {m}_{i}$ matrix of structural coefficients describing the chosen causal structure corresponding to the phenotypes that are available for animal $i$, and ${m}_{i}$ is the number of phenotypes available for animal $i$. It was assumed that ${\mathbf{u}}^{\boldsymbol{*}}\sim N(\mathbf0, \mathbf{A}\otimes{\mathbf{G}}^{*})$, where $\mathbf{A}$ is as defined for the MTM and ${\mathbf{G}}^{*}$ is the SEM additive genetic (co)variance matrix (i.e., it describes variances and covariances of direct genetic effects). Similarly, ${\mathbf{e}}^{\boldsymbol{*}}\sim N(\mathbf0, {\sum }_{\mathrm{i}}^{+}{\mathbf{E}}_{iy})$ where ${\mathbf{E}}_{iy}$ indicates a ${m}_{i}\times {m}_{i}$ matrix with the SEM residual variances corresponding to the phenotypes that were present for animal $i$, and ${m}_{i}$ is the number of phenotypes available for animal $i$. The same model used for SEPBLUP was fitted for SEssGBLUP after replacing the $\mathbf{A}$ matrix by the $\mathbf{H}$ matrix.

The causal phenotypes inferred by the IC algorithm were included as covariates in both SEM models. As SEM accounts for all random variables that simultaneously affect two or more traits, the residual covariances between traits were set to 0 for both models, which allows the structural coefficients to be identifiable [19, 20]. An MCMC chain with the same specifications as used for the MTM was used to estimate the posterior distributions of the SEM parameters. Visual inspection trace plots of posterior distributions and Geweke’s diagnostic [16] applied for MTM were also applied for the SEM to ensure convergence.

### Assessment of accuracy and bias of estimated breeding values

Estimates of the accuracy of estimated breeding values (EBV) for the PBLUP and ssGBLUP models for the data analyzed here were reported in our previous study [9]. Here, the goal was to compare the accuracy obtained based on the SEM to those reported in [9]. First, phenotypes corrected for fixed effects were estimated for all birds using SEBLUP. Then, approximately 10% of the birds (the youngest) had their phenotypes masked, which constituted the validation subset, while the remaining phenotypes were used to train the model (Table 1). The accuracy estimate was the Pearson correlation coefficient between the adjusted phenotypes of the validation subset and their corresponding EBV obtained from the respective models. Bias was assessed using the regression of adjusted phenotypes of the validation subset on their corresponding EBV. In addition to evaluating accuracy and bias of EBV, we also calculated rank correlations between EBV of selection candidates by the two modeling approaches (i.e., between PBLUP and SEPBLUP and between ssGBLUP and SEssGBLUP).

## Results and discussion

### Multiple trait model and inductive causation algorithm analyses

**Fig. 1:**
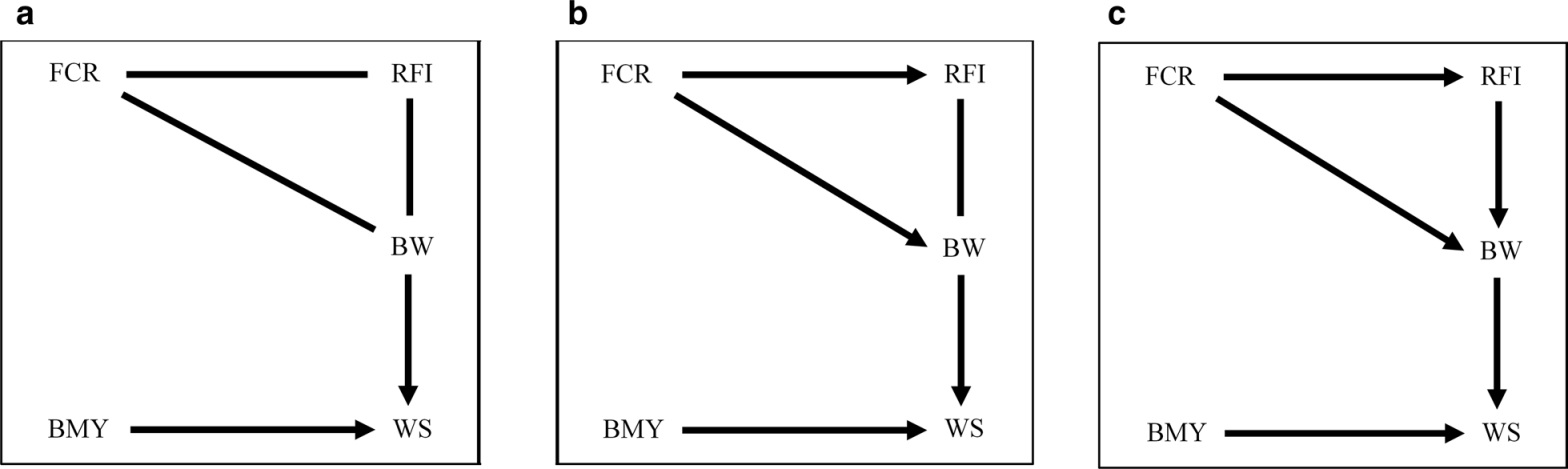
Links between traits detected by the inductive causation algorithm based on 95 (**a**), 90 (**b**) and 85% (**c**) of highest posterior density intervals of the posterior distribution of the residual partial correlation. Links without arrowheads represent associations between traits and those with arrowheads represent causal relationships towards arrowheads. *FCR* feed conversion ratio (kg/kg), *RFI* residual feed intake (kg), *BW* body weight (kg), *BMY* breast meat yield (%), *WS* walking score (1 to 6)

Posterior means of heritabilities and of genetic and residual correlations obtained from the MTM, along with their respective posterior standard deviations (PSD), are in Table 2. These parameters were similar to those reported by Abdalla et al. [9] for the same dataset. HPD intervals of 95, 90, and 85% were applied to detect causal relationships between traits, resulting in the graphs shown in Fig. 1a–c, respectively. Use of the three HPD intervals showed similar links between the traits with PBLUP or ssGBLUP. However, with an HPD of 95%, the direction of the causality was uncovered neither for the connections of FCR with either RFI or BW, nor for the connection between RFI and BW. When the 90% HPD interval was applied, the direction of two (FCR—RFI and FCR—BW) out of the three unknown links was detected (FCR → RFI and FCR → BW). The last unoriented link (RFI—BW) became oriented (RFI → BW) when the 85% HPD interval was used, resulting in a fully directed acyclic diagram (DAG; Fig. 1c).

**Table 2:** Posterior means (Mean) and posterior standard deviations (PSD) of variance components for the multi-trait model and the structural equation model

Component^a^	Multi-trait model		Structural equation model	
	Mean	PSD	Mean	PSD
${\sigma}_{\text{g}}^{2}\text{ FCR}$	0.05	0.01	0.05	0.01
${\sigma}_{\text{g}}^{2}\text{ RFI}$	0.61	0.02	0.53	0.02
${\sigma}_{\text{g}}^{2}\text{ BW}$	0.66	0.05	0.58	0.05
${\sigma}_{\text{g}}^{2}\text{ BMY}$	1.09	0.13	1.09	0.13
${\sigma}_{\text{g}}^{2}\text{ WS}$	0.14	0.02	0.10	0.01
${\sigma}_{\text{e}}^{2} \, {\text{FCR}}$	0.30	0.01	0.31	0.01
${\sigma}_{\text{e}}^{2} \, {\text{RFI}}$	4.26	0.11	3.94	0.09
${\sigma}_{\text{e}}^{2}\,{\text{BW}}$	1.20	0.03	1.24	0.03
${\sigma}_{\text{e}}^{2} \, {\text{BMY}}$	2.94	0.08	2.91	0.08
${\sigma}_{\text{e}}^{2} \, {\text{WS}}$	0.44	0.01	0.37	0.01
${\text{r}}_{\text{g}}\text{ FCR, RFI}$	0.69	0.09	0.62	0.08
${\text{r}}_{\text{g}}\text{ FCR, BW}$	0.17	0.08	0.19	0.09
${\text{r}}_{\text{g}}\text{ FCR, BMY}$	– 0.04	0.04	– 0.08	0.04
${\text{r}}_{\text{g}}\text{ FCR, WS}$	– 0.12	0.07	– 0.08	0.07
${\text{r}}_{\text{g}}\text{ RFI, BW}$	0.13	0.09	0.18	0.09
${\text{r}}_{\text{g}}\text{ RFI, BMY}$	– 0.10	0.08	– 0.19	0.07
${\text{r}}_{\text{g}}\text{ RFI, WS}$	0.07	0.01	0.05	0.01
${\text{r}}_{\text{g}}\text{ BW, BMY}$	0.17	0.09	0.20	0.06
${\text{r}}_{\text{g}}\text{ BW, WS}$	– 0.36	0.06	– 0.32	0.06
${\text{r}}_{\text{g}}\text{ BMY, WS}$	– 0.46	0.13	– 0.29	0.12
${\text{r}}_{\text{e}}\text{ FCR, RFI}$	0.23	0.09	–	–
${\text{r}}_{\text{e}}\text{ FCR, BW}$	– 0.38	0.06	–	–
${\text{r}}_{\text{e}}\text{ FCR, BMY}$	– 0.10	0.03	–	–
${\text{r}}_{\text{e}}\text{ FCR, WS}$	0.03	0.00	–	–
${\text{r}}_{\text{e}}\text{ RFI, BW}$	– 0.17	0.05	–	–
${\text{r}}_{\text{e}} \, \text{ FCR, BMY}$	– 0.07	0.03	–	–
${\text{r}}_{\text{e}} \, {\text{RFI, WS}}$	0.05	0.02	–	–
${\text{r}}_{\text{e}}{\text{ BW, BMY}}$	0.19	0.02	–	–
${\text{r}}_{\text{e}} \, {\text{BW, WS}}$	– 0.15	0.03	–	–
${\text{r}}_{\text{e}} \, {\text{BMY, WS}}$	– 0.06	0.02	–	–
h^2^ FCR	0.14	0.02	0.14	0.02
h^2^ RFI	0.13	0.02	0.12	0.02
h^2^ BW	0.35	0.07	0.32	0.07
h^2^ BMY	0.27	0.04	0.27	0.04
h^2^ WS	0.24	0.04	0.21	0.04

Residual correlations between traits were fixed to 0 for the structural equation models

*FCR* feed conversion ratio (kg/kg), *RFI* residual feed intake (kg), *BW* body weight (kg), *BMY* breast meat yield (%), *WS* walking score (1 to 6)

^a^ ${\sigma}_{\text{g}}^{2}$ genetic variance, ${\sigma}_{\text{e}}^{2}$ residual variance, ${\text{r}}_{\text{g}}$ genetic correlation, ${\text{r}}_{\text{e}}$ residual correlation, *h*^*2*^  narrow sense heritability

### Structural equation model analysis

**Fig. 2:**
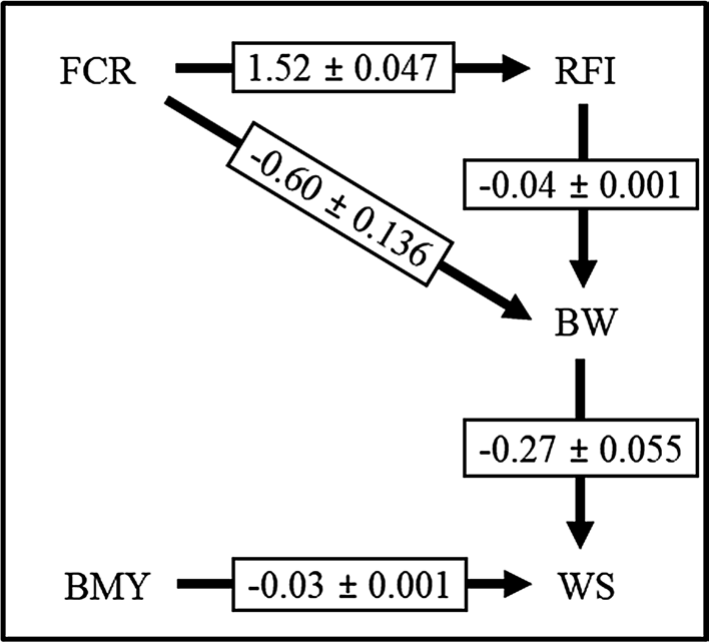
Links and posterior means ± posterior standard deviations of structural coefficients between the studied traits based on results of the inductive causation algorithm based on 85% of highest posterior density intervals of the posterior distribution of the residual partial correlation (Fig. 1c). Links represent causal relationships toward arrowheads. *FCR* feed conversion ratio (kg/kg), *RFI* residual feed intake (kg), *BW* body weight (kg), *BMY* breast meat yield (%), *WS* walking score (1 to 6)

The causal network that was obtained based on the 85% HPD interval (Fig. 1c) was used to fit the SEM. Posterior means of the variance components and of the genetic and residual correlations, along with their respective PSD for the MTM and the SEM, are in Table 2. Links between traits with their respective structural coefficients that were inferred using the SEM are shown in Fig. 2. For some traits, slight differences were observed in the variance component estimates obtained using the SEM versus the MTM. While both FCR and BMY had similar estimates to those from the MTM, genetic variances were lower based on the SEM than based on the MTM for the other three traits. The SEM resulted in smaller estimates of residual variances for all traits, except for FCR and BW. The traits BW and WS also showed different estimates of heritability based on the SEM versus the MTM: from 0.35 ± 0.07 and 0.24 ± 0.04, respectively, for the MTM, to 0.32 ± 0.07 and 0.21 ± 0.04 when the SEM was applied. This suggests that these parameters were over-estimated when causality between traits was ignored. The upstream traits FCR and BMY did not show similar differences in estimates between the SEM and MTM. Such changes in variance component estimates are expected [6, 7] because the traits RFI, BW, and WS are conditioned on at least one of the other two traits, FCR and BMY in the DAG, (see Fig. 1c).

Posterior means of genetic correlations from the SEM also differed from those obtained with the MTM; the SEM resulted in higher posterior means of genetic correlations among all traits, except for the correlations of FCR with RFI (0.69 ± 0.09) and WS (− 0.12 ± 0.07), as well as for the correlation between BMY and WS (− 0.46 ± 0.13). Because genetic effects could be direct or indirect, compared to the MTM, the SEM has the ability to separately identify these types of effects [17]. Although estimates of genetic correlations differed between the two models, these estimates cannot be directly compared, because their definitions differ [6, 17].

Differences in estimates of variance components parameters and of genetic and residual correlations between MTM and SEM have been previously reported. In a study on bovine milk fatty acid, Bouwman et al. [6] reported that unconditioned traits showed lower reductions in estimates of variance components based on the SEM versus the MTM, than the conditioned traits. Similar results were reported by Inoue et al. [7] in their study on the inference of the causal relationship between six meat quality traits in Wagyu beef.

Posterior means of the structural coefficients obtained from the SEM are shown in Fig. 2. All coefficients were negative, except that between FCR and RFI (1.52 ± 0.05). For traits with direct links (i.e., FCR → RFI, FCR → BW, and BMY → WS), the signs of the posterior means of the structural coefficients are expected to be analogous to the sign of the corresponding posterior means of the residual covariances between the same traits obtained from the MTM. The traits FCR and BMY did not have other traits as causal parents and, therefore, the sign of their direct effects on other traits should follow those based on the MTM. Although this is not guaranteed, posterior means of the indirect structural coefficients between traits (i.e., RFI → BW and BW → WS) had the same sign (negative) as their counterpart residual covariances in the MTM. However, the converse can also be true, as indirect structural coefficients are inferred from a conditional association and the residual covariances are marginal associations [5].

Results indicated that RFI is positively affected by FCR, which was inferred at a magnitude of 1.52 ± 0.05 (Fig. 2). The connection between these two feed efficiency traits has previously been reported for turkeys [12, 21] and other species, e.g. [22, 23]. The main difference between them is that FCR is expressed as a ratio of feed intake and growth rate, while RFI is a linear index of feed intake and growth rate. However, as a ratio of two component traits, genetic selection for FCR may not account for how selection is working on each trait directly [24]. Moreover, FCR is not independent of production traits and also does not account for them. Such problems are not encountered with RFI but animals that have favorable RFI could grow slowly [25]. Regardless, Case et al. [12] compared FCR and RFI in a turkey population and reported that both could be integrated into a turkey selection index, but RFI may be advantageous because it is more independent of performance traits than FCR. In general, both traits are important to improve feed efficiency and both can be considered as “expensive-to-measure” phenotypes. Investigating the direction and the magnitude of the association between FCR and RFI seems critical for selection indexes that aim at improving simultaneous multiple traits through genetic selection.

Estimates of the structural coefficients of FCR with BW (− 0.60 ± 0.14) and of RFI with BW (− 0.04 ± 0.00) were both negative. Slightly lower genetic correlations of FCR (0.65 ± 0.05) and RFI (0.09 ± 0.06) with BW have been reported for turkeys [12] compared to our estimates (0.65 ± 0.05 and 0.09 ± 0.06, respectively). Although the results of this study suggest that FCR has more influence on BW than RFI based on a higher estimate of the structural coefficient, the posterior distribution for the structural coefficient of RFI on BW showed a relatively wider range (SD = 2.51; Table 1) and, therefore, its true value could be quite different from the posterior mean. Moreover, RFI is expected to increase by 1.52 for each unit increase in FCR, which implies that RFI is expected to improve as a result of selection for FCR. Thus, selection on either trait may have similar effects on BW. Although RFI is calculated by adjusting for BW, it may not guarantee that the genetic correlation is zero. Thus, independence of RFI from BW could be confounded by the change in growth rate [26, 27]. Depending on the ultimate goal of the breeding program, the causal effects between BW and these two feed efficiency traits suggest favorable conditions for the joint genetic improvement of these three traits. Selection for lower FCR is expected to lead to better RFI and BW. It is well known that RFI and FCR are genetically strongly related [9, 12] and have weak relationships with BW, given that efficiency traits are implemented to reduce feed consumption and increase/maintain body weight gain. Uncovering causal effects provides insights and hypotheses about how causal relationships between traits may contribute to better selection decisions.

Based on the estimated structural coefficients, BW and BMY are expected to influence WS negatively, which indicates that for each kg of BW and for each 1-unit of BMY, WS is expected to drop by 0.27 ± 0.06 and 0.03 ± 0.00, respectively (Fig. 2). These three phenotypes are key traits in turkey breeding programs. Body weight and BMY are among the most important phenotypes in turkey populations raised for meat, while adequate walking ability ensures that birds maintain good health and fitness. Genetically, BW and BMY are unfavorably correlated with WS (e.g., [9, 26, 27]), which could be due to the relatively faster rate of improvement in BW and BMY as a result of direct selection for these traits, than the rate of improvement in muscles and bones of the legs. The close relationship between body size and the relative proportion of body parts is expected to cause a decline in the proportion of leg muscle and bone as BW and BMY increase [28, 29]. This disproportionate relationship is associated with genetic increases in BW [30].

Nestor et al. [28] reported that leg abnormalities starting at the age of 16 weeks has increased in turkeys as a result of years of selection for body and breast meat. Our study shows that interventions (e.g., management practices [5, 17]) on BW or BMY may block the indirect genetic effects through them on WS. Previous studies have reported that walking ability can be improved by genetic selection without negatively affecting BW [29, 31]. From a six-generation study, Emmerson et al. [29] reported that a single-trait selection for increased shank width resulted in improved walking ability in large-bodied turkeys, while BW continued to increase. These findings are consistent with results reported in Nestor et al. [32] from two selection experiments that aimed at increasing the relative proportions of leg muscle and bone in turkeys. Although the magnitude of the causal coefficient of BMY on WS was low (− 0.03 ± 0.001), the influence of BMY on WS could be strong, given that the phenotype of BMY is large (24.37 ± 2.33; Table 1). The influence of BMY on waking ability could also be due to a physical pressure of the breast on the legs and, as a result, on walking ability.

### Accuracy and bias

Estimates of prediction accuracy and bias of EBV from MTM (PBLUP and ssGBLUP; Abdalla et al. [9]) and SEM (SEPBLUP and SEssGBLUP) are in Tables 3 and 4, respectively. As expected, there was no difference in the accuracy of EBV for the upstream traits (FCR and BMY) between MTM and SEM since they were unconditioned in the SEM. However, estimates of the accuracy of EBV for the three downstream traits (RFI, BW and WS) were higher with SEM than with MTM. The accuracy estimates for RFI, BW, and WS increased by approximately 5, 6, and 19%, from 0.21, 0.36 and 0.26 under PBLUP to 0.22, 0.38 and 0.31, respectively, under SEPBLUP. Similar increases in accuracy were also observed between ssGBLUP and SEssGBLUP, with increases by approximately 7, 5, and 20% for RFI, BW, and WS, respectively. Estimates of bias showed slight differences between the MTM and SEM models, with BW and WS having the largest differences (Table 4). The regression coefficients for BW were 0.86 ± 0.03 and 0.89 ± 0.03 with SEPBLUP and SEssGBLUP, respectively, and 0.82 ± 0.03 and 0.83 ± 0.03 with PBLUP and ssGBLUP, respectively. With SEM, estimates of bias for WS also tended to reach 1 for SEPBLUP (0.73 ± 0.04 to 0.78 ± 0.04) and SEssGBLUP (0.75 ± 0.04 to 0.82 ± 0.04).

**Table 3:** Accuracy of estimated breeding values for the studied traits based on the pedigree-based best linear unbiased prediction (PBLUP), single-step genomic best linear unbiased prediction (ssGBLUP), structural equation PBLUP (SEPBLUP), and the structural equation ssGBLUP (SEssGBLUP)

Trait	PBLUP	SEPBLUP	ssGBLUP	SEssGBLUP
Feed conversion ratio	0.29	0.29	0.38	0.38
Residual feed intake	0.21	0.22	0.27	0.29
Body weight	0.36	0.38	0.40	0.42
Breast meat yield	0.30	0.30	0.37	0.37
Walking score	0.26	0.31	0.30	0.36

**Table 4:** Bias of estimated breeding values for the studied traits based on pedigree-based best linear unbiased prediction (PBLUP), single-step genomic best linear unbiased prediction (ssGBLUP), structural equation PBLUP (SEPBLUP), and the structural equation ssGBLUP (SEssGBLUP))

Trait	PBLUP	SEPBLUP	ssGBLUP	SEssGBLUP
Feed conversion ratio	0.94 ± 0.17	0.94 ± 0.17	0.95 ± 0.17	0.95 ± 0.17
Residual feed intake	0.79 ± 0.12	0.79 ± 0.12	0.80 ± 0.12	0.81 ± 0.12
Body weight	0.82 ± 0.03	0.86 ± 0.03	0.83 ± 0.03	0.89 ± 0.03
Breast meat yield	1.41 ± 0.21	1.40 ± 0.20	1.38 ± 0.21	1.38 ± 0.20
Walking score	0.73 ± 0.04	0.78 ± 0.04	0.75 ± 0.04	0.82 ± 0.04

Our results indicate that the accuracy of EBV could be enhanced by incorporating causal relationships between traits. When traits share causal effects among them, breeding strategies based only on MTM analysis could lead to wrong selection decisions [5], which would result in slower selection progress than expected and the indirect effects between traits would be ignored. As discussed in Abdalla et al. [9], in turkey breeding programs selection is not expected to be among selection candidates from different generations. As a result, the biases in EBV can be neglected. However, biases should be adjusted for and carefully examined when selection candidates are from different generations. Incorporating the causal structures may slightly reduce the biases in EBV, but other techniques should be used to account for any potential bias of predictions that such models may yield.

The MTM and SEM resulted in similar rank correlations of EBV for all traits for both pedigree-based and genomic analyses. While the EBV for the upstream traits (FCR and BMY) showed complete agreement between MTM and SEM, with rank correlations of 1.0, as expected, rank correlations for RFI, BW, and WS were 0.88, 0.93 and 0.86, respectively. Although RFI was conditioned by only one trait (FCR), it showed a lower rank correlation of EBV than BW (conditioned by FCR and RFI) and similar to WS (conditioned by all traits). This could be due to the number of selection candidates for each trait. Abdalla et al. [32] compared rank correlations between linear and threshold animal models using different numbers of selection candidates and reported that the similarity in rankings increased as the number of selection candidates increased. Depending on breeding plans, the highest-ranking animals are more likely to be selected for breeding. Hence, selection of the best parents to produce the next generation may not be as accurate when ignoring causal relationships between traits.

In this study, males were phenotyped for all traits whereas females were only phenotyped for body weight and walking ability. As a result, analysis of only the female population would be limited to the connection between body weight and walking ability. Nevertheless, including data on females enhances the accuracy of EBV and of estimates of genetic parameters [1]. This was even more beneficial in this study because a large portion of the genotyped animals were females, which should improve the identification of the relationships between animals leading to a higher predictive ability for the genetic merit for the selection candidates [33]. It should be noted that EBV obtained from SEM are adjusted for both systematic effects and structural coefficients. In practice, it is quite important for turkey breeders to investigate the causality network between all traits in females and perform more accurate selection decisions, including traits that are not measured in the female population.

Descriptive statistical models that lack information about causal relationships among traits may not detect the changes in dependent phenotypes due to external interventions on causal parents traits [5, 17]. This is where the inevitable ambiguity of relying on correlation information in making inferences about the association among a set of traits lies. One of the most common strategies to assess dependencies and causal effects of interest is conditioning [34]. This can be done, as in this study, by using covariate adjustment to include realized values of explanatory variables in the linear predictor of a statistical model [35]. Investigating the phenotypic networks among physiological traits that may exert causal effects on each other is absolutely relevant for accelerating breeding programs. For instance, increased feed intake in high producing dairy cows enhances liver blood flow and metabolism, which in turn, influences the concentrations of circulating critical innate reproductive hormones, such as estradiol and progesterone, leading to negative effects on their reproductive performance [36]. Thus, ignoring the structural relationships between traits and applying selection based only on MTM models may result in slower genetic progress in economically important traits.

## Conclusions

In this study, we investigated the functional relationships between five traits in a turkey line and their effects on the accuracy of EBV under pedigree-based and single-step genomic evaluation models. Applying a 95% posterior density interval for the posterior distribution of the residual partial correlation uncovered links between the traits, but some of them were not directed. A fully DAG was obtained with the use of a narrower (85%) HPD interval. The SEM based on this graph allowed the potential indirect genetic effects between the traits and their magnitudes to be estimated. Differences in the estimates of genetic variance and the ranks of the EBV of selection candidates between the MTM and the SEM suggest that the rate of genetic improvement for the breeding goal could be reduced if selection decisions are based only on EBV derived from the MTM. These findings were supported by the higher prediction accuracies for conditioned traits compared to assuming no connections between phenotypes. Our results also suggest that interventions on BW or BMY may block the indirect genetic effects through them on WS. Using structural equation models can accelerate genetic progress and enhance the accuracy of EBV for the selection of turkey candidates in both pedigree-based and single-step genomic models.

## Data Availability

Data that support the findings of this study are available from Hybrid Turkeys upon reasonable request, but restrictions apply to the availability of these data, which were used under license for the current study, and thus are not publicly available.
